# Effectiveness of Interprofessional Consultation-Based Interventions for Delirium: A Scoping Review

**DOI:** 10.1177/07334648211018032

**Published:** 2021-06-02

**Authors:** Caitlin Monaghan, Grace Martin, Jason Kerr, Mary-Lynn Peters, Judith Versloot

**Affiliations:** 1 Trillium Health Partners, Mississauga, Ontario, Canada; 2 University of Toronto, Ontario, Canada; 3 McMaster University, Hamilton, Ontario, Canada

**Keywords:** delirium, interprofessional team, older adults, consultation, inpatient

## Abstract

**Background:**

Interprofessional geriatric consultation teams and multicomponent interventions are established models for delirium care. They are combined in interprofessional consultative delirium team interventions; however, insight into this novel approach is lacking.

**Objective:**

To describe the effectiveness and core components of consultation-based interventions for delirium.

**Method:**

Ovid MEDLINE, EMBASE, PsycINFO, CINAHL, and ProQuest. Data on core intervention components, outcomes, facilitators, and barriers were extracted.

**Results:**

10 studies were included. Core intervention components were systematic delirium screening, ongoing consultation, implementation of non-pharmacologic and pharmacological interventions, and staff education. Of the included studies, 1/6 found a significant reduction in delirium incidence, 1/2 a reduction in delirium duration, and 2/3 found a reduction in falls. Facilitators and barriers to implementation were discussed.

**Conclusion:**

There was consistency in team structure and core components, however intervention operationalization and effectiveness varied widely. There is some evidence that this model is effective for reducing delirium and its sequelae.

## Introduction

Delirium is an acute change in mental state that develops suddenly, often goes unrecognized, and increases an individual’s risk of adverse events including death, a longer hospital stay, and permanent cognitive decline (Hullick et al., 2018; Piotrowicz et al., 2018). It is a highly prevalent condition, seen in 18% to 50% of patients upon admission and incident in 11% to 82% during hospitalization, with older, medically complex populations being the most at-risk (Rubin et al., 2018). Current evidence suggests that 30% to 40% of delirium cases are preventable, highlighting the importance of targeted, in-hospital interventions to prevent delirium, its downstream morbidities, and associated health and economic costs (Inouye et al., 1999, 2014). Accordingly, there is a widespread effort underway across the globe to better address delirium in hospitalized patients (Inouye et al., 2014).

Interprofessional geriatric inpatient consultation teams (IGCTs, also referred to as inpatient geriatric consultation services, geriatric assessment teams, or geriatric liaison teams) are recognized as a promising model of geriatric care. They are mobile teams that conduct comprehensive assessment of older inpatients on non-geriatric wards and provide recommendations for patient care to unit staff (Trogrlić et al., 2015). This model allows for a higher number of patients to be seen by geriatric specialists in settings where the capacity of acute geriatric units is insufficient to accommodate the volume of admitted older adults. Although widely implemented and well-liked by patients, clinicians, nurse managers and decision-makers, a recent scoping review found that these teams are highly heterogeneous in structure and process, and evidence on their effectiveness is inconclusive (Trogrlić et al., 2015).

IGCTs often address delirium by providing recommendations for prevention or management (Deschodt et al., 2016), however current systematic reviews and clinical guidelines recommend multicomponent interventions targeting modifiable risk factors to address delirium in at-risk patients (Hshieh et al., 2015; Inouye et al., 2014; Reston & Schoelles, 2013). These approaches often include a combination of regular screening for delirium, geriatric specialist consultation, delirium related education for unit staff, non-pharmacological and pharmacological protocols, and interprofessional collaboration for comprehensive delirium management (Mudge et al., 2013). The Hospital Elder Life Program (HELP) is the original evidence-based multicomponent intervention targeting delirium, which uses an interdisciplinary team and trained volunteers to implement interventions and educate clinical peers about geriatric care. The program has been found to reduce delirium incidence, length of hospital stay, and patient complications. In addition, several studies have found these multicomponent interventions to be cost-effective (Akunne et al., 2012, 2014), making them an attractive strategy for practitioners and policymakers to consider.

Some interventions have used a modified version of the IGCT model to deliver multicomponent strategies targeting delirium, which we refer to as interprofessional consultative delirium teams throughout this review. These teams provide consultation to older inpatients and make recommendations for delirium care to unit staff. Insight into the structures, care processes and effectiveness of such teams is currently lacking in the literature. Therefore, the aim of this review is to identify the core components of interprofessional consultative delirium team initiatives, describe their effectiveness on reducing delirium incidence and related outcomes, and summarize facilitators and barriers to their implementation.

## Method

We conducted a scoping review to identify existing academic literature pertaining to interprofessional consultative delirium teams in March 2020. This study followed a methodological framework for scoping reviews (Arksey & O’Malley, 2005; Levac et al., 2010) in addition to PRISMA guidelines (Tricco et al., 2018).

### Identify the Research Question

A broad research question with a clearly articulated scope of inquiry and outcomes of interest was developed to facilitate a comprehensive range of coverage (Levac et al., 2010): “What is the current state of academic literature related to the implementation of inpatient interprofessional consultative delirium teams and their effectiveness for reducing delirium and its related morbidities?”

### Identify and Select Relevant Studies

Two strategies were used to obtain studies: (a) a rigorous search of electronic databases, including Ovid, EMBASE, PsycINFO, CINAHL, and ProQuest; and (b) citation mining from included studies. The search strategy was undertaken in English in collaboration with a Health Sciences Librarian (see Table 1 and Supplemental Appendix A).

**Table 1.** Sample Key Word Search Strategy (ProQuest).

Key words	Exact(“delirium”) AND Exact(“frail elderly” OR “aged, 80 over” OR “aged, 80 AND over”) AND Exact(“patient care team” OR “hospital rapid response team” OR “patient care planning” OR “geriatric nursing” OR “interdisciplinary approach” OR “referral and consultation” OR “postoperative care” OR “geriatric assessment” OR “multidisciplinary practices” OR “interdisciplinary team work”)
Limits	(English language and yr= “2000 -Current)

To be included, studies needed to meet the following inclusion criteria: published in English between January 2000 and March 2020; evaluated the effectiveness of a hospital-based intervention to reduce delirium or improve outcomes for patients with delirium (e.g., delirium incidence/severity/duration, LOS); included an interprofessional team and mobile inpatient consultation component; and used a validated tool to detect delirium. These criteria were set to capture a range of existing care models while ensuring a basic standard of quality and focus on relatively current literature. Studies were excluded if they did not either target patients with delirium or measure delirium as an outcome, consultations did not occur in-person (e.g., unit-based, E-health, tele-health), were published protocols with no evaluation, or were comprehensive geriatric assessment-based interventions that do not specifically address delirium. It should be noted that many inpatient delirium-based interventions, including some foundational programs such as the HELP, did not include a consultative component and thus did not meet the inclusion criteria for this review.

**Figure 1.**
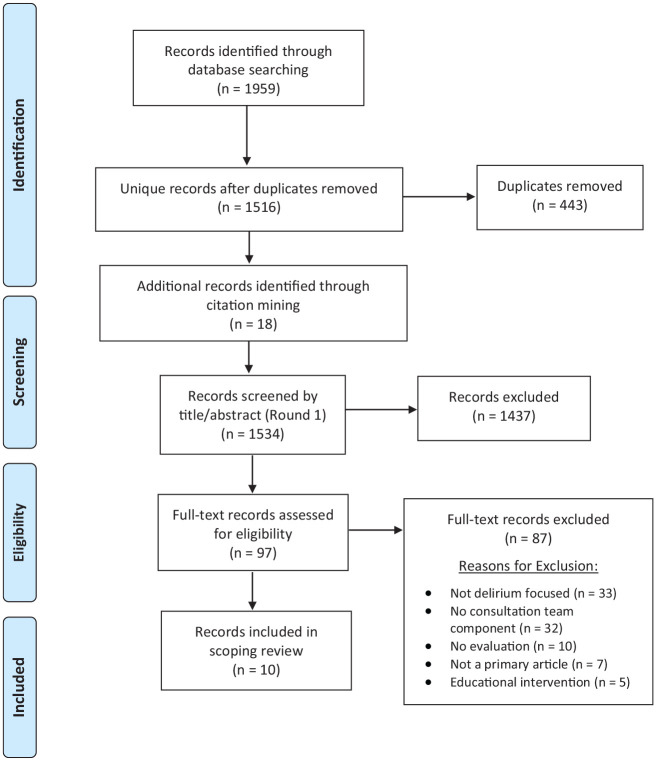
PRISMA flow diagram of literature search and screen process.

An initial eligibility assessment, title and abstract screen, and full text review was conducted independently by two reviewers (C.M. & G.M.), with a third reviewer available to resolve disagreement (J.V.). Two reviewers (C.M. & G.M.) applied the inclusion and exclusion criteria throughout the full text review, and reasoning for inclusion or exclusion was documented. The third reviewer (J.V.) reviewed the final selection of articles and verified the reasoning for inclusion or exclusion. Figure 1 details the complete screening process. In total, *n* = 10 records were included in the review.

### Data extraction and thematic analysis

Data extraction was performed independently by two reviewers (C.M. & G.M.) and verified by a third (J.V.) to ensure accuracy. Data on the type of study, interprofessional team composition, intervention components, target population, delirium assessment method, and barriers and facilitators to implementation were extracted and charted. The quantity and heterogeneity of the literature was described. Key components of consultative delirium team models were analyzed and summarized, emerging themes were discussed, and knowledge gaps and areas for future research were identified.

## Results

A total of 1,516 unique articles were identified and screened (see Figure 1). Ten articles were included in the review, all of which were interventions delivered by an interprofessional consultative team to manage delirium in hospitalized patients.

### Study Designs, Settings, and Target Populations

A range of study designs were applied to evaluate the included interventions, and all were conducted in North America or Europe. The interprofessional consultation teams were implemented on a variety of in-hospital units, and most targeted older adults (see Table 2 for details).

**Table 2.** Description of Included Studies and Their Respective Interprofessional Consultative Delirium Teams, Reverse Chronology by Publication Date.

Author(s), year	Study design, location	Target population	Delirium detection/screening	Delirium outcome (incidence, duration, or severity) *compared to usual care
Bryant et al. (2019)	Retrospective cohort study, USA	Patients 65 years or older admitted to a trauma service	Patients were screened with the CAM* at least once throughout hospitalization	*Incidence*: significant decrease in intervention group (21.6% vs. 12.5%; *p* = .04), which remained significant after adjustment (OR = 0.44; 95% CI = [0.22, 0.88]; *p* = .02)
Tarazona-Santabalbina et al. (2018)	Retrospective cohort study, Spain	Patients 70 years or older admitted for elective colorectal cancer surgery	Patients were screened with the CAM by physicians or nurses every 8 hr	*Incidence*: significant decrease in intervention group (11.3% vs. 29.2%; *p* < .001)
Ferguson et al. (2018)	Retrospective cohort study, USA	Patients on all hospital nursing units at a tertiary care hospital	A delirium risk identification form was completed by unit staff at time of admission. The CAM or CAM-ICU* was conducted on patients identified as at-risk twice per day	N/A
Angel et al. (2016)	QI project, USA	Older adults	The CAM-ICU was completed 2+ times daily by unit staff	N/A
Babine et al. (2013)	Evidence-based practice project, USA	Patients 70 years or older at risk for falls, taking a Beers list medication, or prescribed a sedative	The CAM was completed by unit staff on admission, every 12 hr, and as warranted with suspicion of cognitive changes	N/A
Hempenius et al. (2013)	Multicentre RCT, The Netherlands	Cancer patients 65 or older undergoing elective surgery for a solid tumor	The DOS* was completed by a ward nurse three times per day for up to 10 days postoperatively. Delirium Rating Scale—Revised-98 was used to assess delirium severity	*Incidence, severity, and duration*: no difference
Deschodt et al. (2012)	Parallel-group controlled trial, Belgium	Patients 65 years or older admitted with hip fracture	Three nurses blinded to study outcomes performed all assessments, CAM, DI*, and MMSE* were collected preoperatively and on postoperative days 1, 3, 5, 8, and 15	*Incidence*: significant decrease in intervention group (37.2% vs. 53.2%, *p* = .4, OR = 1.92, 95% CI = [1.04, 3.54]), non-significant after adjustment (*p* = .07, OR = 1.79, 95% CI = [0.95, 3.37]). *Severity, duration*: no difference
Cole et al. (2002)	RCT, Canada	Patients 65 or older with a positive CAM within first week of hospitalization	The MMSE and DI were conducted 3 times in the first week of hospitalization, weekly thereafter	*Severity*: no difference
Marcantonio et al. (2001)	Prospective randomized trial, USA	Patients 65 and older admitted emergently for surgical repair of hip fracture	A blinded research interviewer conducted the CAM upon intake and the MMSE, DSI*, MDAS*, and CAM daily	*Incidence*: significant decrease in cumulative incidence in intervention group (32% vs. 50%, *p* =. 04; RR = 0.64; 95% CI = [0.18, 0.89]). *Severity*: cumulative incidence of severe delirium significantly reduced in the intervention group, (12% vs. 29%, *p* = .02; RR = 0.4; 95% CI = [0.19, 0.89]). Neither were significant after adjustment
Milisen et al. (2001)	Prospective, longitudinal sequential design, Belgium	Patients with a traumatic hip fracture hospitalized within 24 hr of surgery	The NEECHAM* was conducted by unit staff daily, if positive the CAM was used to verify delirium on postoperative day 1, 3, 5, 8, and 12	*Incidence*: no difference between groups. *Severity, duration*: a significant reduction in severity (*p* = .0152) and duration of delirium (*p* = .03; median = 1 day vs. 4 days) in intervention group

*Note.* CAM = confusion assessment method; OR = odds ratio; CI = confidence interval; CAM-ICU = confusion assessment method–intensive care unit; DOS = Delirium Observation Scale; DI = Delirium Index; MMSE = Mini-Mental State Examination; DSI = Delirium Symptom Interview; MDAS = Memorial Delirium Assessment Scale; NEECHAM = Neelon and Champagne Confusion Scale.

### Interprofessional Team Composition

Interprofessional consultation teams were each composed of at least two distinct professions providing patient consultation or recommendations for care to unit staff. Most teams included a physician and a nurse, with at least one specialized in geriatrics. Eight included a geriatrician (Babine et al., 2013; Bryant et al., 2019; Cole et al., 2002; Deschodt et al., 2012; Gorski et al., 2017; Hempenius et al., 2013; Marcantonio et al., 2001; Milisen et al., 2001; Tarazona-Santabalbina et al., 2019), and three a psychiatrist (Angel et al., 2016; Cole et al., 2002; Ferguson et al., 2018). Four teams included a clinical nurse specialist (Angel et al., 2016; Babine et al., 2013; Ferguson et al., 2018; Tarazona-Santabalbina et al., 2019), two a registered nurse (Cole et al., 2002; Deschodt et al., 2012), and three a geriatric nurse (Hempenius et al., 2013; Milisen et al., 2001; Tarazona-Santabalbina et al., 2019). Additional disciplines included across the teams were volunteers (Babine et al., 2013), allied health professionals (including representatives from social work, occupational therapy, physiotherapy, and speech and language pathology) (Bryant et al., 2019; Deschodt et al., 2012), clinical educators (Ferguson et al., 2018), and an orthopedics team (Marcantonio et al., 2001). No teams included a pharmacist.

### Intervention Components

Five key intervention components were found to be included across all studies: systematic cognitive screening and identification of delirium, interprofessional team consultation, implementation of non-pharmacological strategies, medication management, and staff education and distribution of educational materials (see Table 3). A detailed description of each component is below.

**Table 3.** Common Intervention Components for Interprofessional Consultative Delirium Teams, Reverse Chronology by Publication Date.

Intervention component (no. of studies)	Bryant	Tarazona	Ferguson	Angel	Babine	Hempenius	Deschodt	Cole	Marcantoni	Milsen
**Systematic cognitive screening for delirium**										
2+ times/day (5)		X	X	X	X	X				
Daily (2)									X	X
Every other day (2)							X	X		
Once (1)	X									
**Medication management**										
Medication management protocol/guidelines (4)		X		X		X				X
Recommendations for medication management upon consultation (9)	X		X	X	X	X	X	X	X	X
**Education of unit staff**										
Mandatory educational sessions (5)	X			X	X	X	X			
Training on validated screening tools (7)			X	X	X	X	X		X	X
Training of “delirium champions” (2)	X									X
Distribution of educational or instructive materials (protocols, posters) (7)	X	X	X			X		X	X	X
Ongoing education upon consultation (7)			X	X	X	X		X	X	X
**Consultation**										
Daily rounds/visits (4)		X				X		X	X	
Weekday rounds/visits (2)				X	X					
Weekly rounds/visits (1)			X							
Single follow-up visit (1)							X			
Available as needed (2)	X									X
**Proactive identification of high-risk patients**										
Yes (10)	X	X	X	X	X	X	X	X	X	X
No (0)										
**Interprofessional team composition**										
Geriatrician (8)	X	X			X	X	X	X	X	X
Psychiatrist (3)			X	X				X		
Clinical nurse specialist (4)		X	X	X	X					
Registered nurse (2)							X	X		
Geriatric nurse (3)		X				X				X
Allied health (1)	X									
**Non-pharmacological strategies**										
Mobility (7)	X		X	X	X	X		X	X	
Orientation (6)	X			X	X	X		X	X	
Sensory adaptation (6)			X	X	X	X		X	X	
Hydration (5)		X	X		X	X			X	
Nutrition (5)	X		X		X	X			X	
Sleep (5)			X	X	X	X		X		
Bowel/bladder elimination (5)	X		X	X		X			X	

*Note.* X indicates that the component was included in the intervention, blank indicates it was not.

### Systematic Cognitive Screening and Identification of Delirium

The systematic screening and identification of delirium was often performed two or more times per day (Angel et al., 2016; Babine et al., 2013; Ferguson et al., 2018; Hempenius et al., 2013; Tarazona-Santabalbina et al., 2019) or daily (Marcantonio et al., 2001; Milisen et al., 2001), with the remaining interventions screening every other day (Cole et al., 2002; Deschodt et al., 2012) or once (Bryant et al., 2019). It was typically conducted by unit staff as a part of daily nursing care using the Confusion Assessment Method (CAM), a validated instrument and diagnostic algorithm for identifying delirium that is the most widely used standardized delirium instrument for clinical and research purposes (Wei et al., 2008). Tools including the Mini-Mental State Examination (MMSE), Delirium Index (DI), CAM-ICU (an adaptation of the CAM designed to detect delirium in intensive care patients), and Memorial Delirium Assessment Scale were used alongside the CAM. Two studies did not use the CAM: one implemented the MMSE in combination with the DI (Cole et al., 2002), and the other used the Delirium Observation Scale followed by the Delirium Rating Scale—Revised-98 to measure severity of delirium (Hempenius et al., 2013).

#### Interprofessional Team Consultation

Members of the consultative team would provide patient-specific recommendations for preventing or managing delirium to unit staff through ongoing consultation. This was conducted at various frequencies; most teams conducted daily (Cole et al., 2002; Hempenius et al., 2013; Marcantonio et al., 2001; Tarazona-Santabalbina et al., 2019), weekday (Angel et al., 2016; Babine et al., 2013), or weekly rounds (Ferguson et al., 2018). One team provided consults from each specialty (geriatrics, allied health, etc.) as needed (Bryant et al., 2019), another had check-ins as needed (Milisen et al., 2001), and the remaining team consulted only once to address any problems and ensure that previously made recommendations were being implemented (Deschodt et al., 2012).

#### Non-pharmacological strategies

Nonpharmacological interventions were included in all but two studies (Deschodt et al., 2012; Milisen et al., 2001). The most frequently implemented strategies were related to mobility (e.g., ambulation, range-of motion exercises, keeping mobility devices within reach) (Angel et al., 2016; Babine et al., 2013; Bryant et al., 2019; Cole et al., 2002; Ferguson et al., 2018; Hempenius et al., 2013; Marcantonio et al., 2001); orientation (e.g., provision of a clock and calendar, encouraging family visits, allowing personal items from home) (Angel et al., 2016; Babine et al., 2013; Bryant et al., 2019; Cole et al., 2002; Hempenius et al., 2013; Marcantonio et al., 2001); and sensory adaptation (e.g., preventing sensory deprivation or overload, appropriate use of glasses and hearing aids) (Angel et al., 2016; Babine et al., 2013; Cole et al., 2002; Ferguson et al., 2018; Hempenius et al., 2013; Marcantonio et al., 2001). Other common strategies were related to hydration (e.g., assistance with fluids, building steps to prevent dehydration into the protocol) (Babine et al., 2013; Ferguson et al., 2018; Hempenius et al., 2013; Marcantonio et al., 2001; Tarazona-Santabalbina et al., 2019); nutrition (e.g., assistance with food consumption, asking caregivers to provide dentures) (Babine et al., 2013; Bryant et al., 2019; Ferguson et al., 2018; Hempenius et al., 2013; Marcantonio et al., 2001); sleep (e.g., help with relaxation, sleep hygiene, preventing an impaired sleep-wake cycle, draw curtains to allow sunlight during daylight hours); and bowel/bladder functioning (e.g., assistance with toileting, preventing constipation and urinary retention) (Angel et al., 2016; Bryant et al., 2019; Ferguson et al., 2018; Hempenius et al., 2013; Marcantonio et al., 2001).

#### Medication management

All interventions included a component to address the use of medication, either through patient-specific recommendations for medication management upon consultation (Angel et al., 2016; Babine et al., 2013; Bryant et al., 2019; Cole et al., 2002; Deschodt et al., 2012; Ferguson et al., 2018; Hempenius et al., 2013; Marcantonio et al., 2001; Milisen et al., 2001), and/or the provision of medication management/dosing protocols related to delirium and pain medications (Angel et al., 2016; Hempenius et al., 2013; Milisen et al., 2001; Tarazona-Santabalbina et al., 2019). Of the 10 included studies, 9 included a component to review and minimize medications that contribute to delirium (e.g., benzodiazepines, anticholinergics, antihistamines) (Angel et al., 2016; Babine et al., 2013; Bryant et al., 2019; Cole et al., 2002; Deschodt et al., 2012; Ferguson et al., 2018; Hempenius et al., 2013; Marcantonio et al., 2001; Milisen et al., 2001). Five studies addressed pain by optimizing non-opioid and opioid analgesia (Angel et al., 2016; Ferguson et al., 2018; Marcantonio et al., 2001; Milisen et al., 2001; Tarazona-Santabalbina et al., 2019) with one using standardized order sets for pain medications (Milisen et al., 2001). Two studies included guidelines regarding the use of neuroleptics to treat delirium-related behaviors such as agitation (Angel et al., 2016; Marcantonio et al., 2001), with one providing medication dosing guides for hospital physicians (Angel et al., 2016).

#### Staff education and distribution of educational materials

All studies provided training on how to prevent, identify, and manage delirium to unit staff involved in intervention delivery (Angel et al., 2016; Babine et al., 2013; Deschodt et al., 2012; Ferguson et al., 2018; Hempenius et al., 2013; Marcantonio et al., 2001; Milisen et al., 2001). The modality and intensity of education varied widely, ranging from; mandatory educational sessions (Angel et al., 2016; Babine et al., 2013; Bryant et al., 2019; Deschodt et al., 2012; Gorski et al., 2017; Hempenius et al., 2013), standing meetings and real-time education as a part of patient consults (Angel et al., 2016; Babine et al., 2013; Cole et al., 2002; Ferguson et al., 2018; Hempenius et al., 2013; Marcantonio et al., 2001; Milisen et al., 2001), and training of unit champions who remained on units and were available to assist fellow unit staff (Bryant et al., 2019; Milisen et al., 2001).

In addition, educational materials related to the delirium interventions were frequently distributed to engaged units. Protocols/checklists for implementation of delirium prevention and management strategies were distributed to unit staff (Bryant et al., 2019; Ferguson et al., 2018; Gorski et al., 2017; Marcantonio et al., 2001; Tarazona-Santabalbina et al., 2019), and posters that describe symptoms of delirium or highlight the importance of accurate and early detection of delirium were placed on units (Hempenius et al., 2013; Milisen et al., 2001).

### Process and Clinical Outcomes

See Tables 2 and 4 for an overview of key process and clinical outcomes.

**Table 4.** Clinical Outcomes Measured to Evaluate Consultative Delirium Team Success, Reverse Chronology by Publication Date.

Author	Key clinical outcomes evaluated				
	Delirium incidence	Delirium severity	Delirium duration	LOS	Falls
Bryant	⇩	–	–	–	–
Tarazona-Santabalbina	⬇	–	–	=	–
Ferguson	–	–	–	–	⬇
Angel	–	–	–	⬇	Study underpowered
Babine	–	–	–	–	⬇
Hempenius	=	=	–	=	–
Deschodt		=	=	=	–
Cole	–	=	–	=	–
Marcantonio	⇩	⇩	–	=	–
Milisen	=	⬇	⬇	=	–

*Note.* The bold arrow indicates a statistically significant reduction in the intervention group compared with the control group; The non-bolded arrow indicates a non-significant reduction in the intervention group compared with the control group; = indicates no difference between groups; and – indicates that the outcome was not considered. LOS = length of hospital stay.

#### Length of hospital stay

Length of hospital stay (LOS), was evaluated by 7 of the 10 studies (Angel et al., 2016; Deschodt et al., 2012; Hempenius et al., 2013; Marcantonio et al., 2001; Milisen et al., 2001). One found a significant difference, where the average LOS of the intervention group decreased significantly from 8.5 to 6.5 days (*p* = .001) after implementation (Angel et al., 2016), and the remaining studies found no difference in LOS between groups.

#### Delirium incidence, severity and duration

Seven studies reported on delirium incidence, duration, or severity, using a variety of assessment methods and obtaining various results. Of the six that evaluated delirium incidence as an outcome, only one found a significant reduction in the intervention group, three found non-significant reductions, and the remaining two found no differences between groups. Information on delirium severity and duration can be found in Tables 2 and 4.

#### Falls

Two (Babine et al., 2013; Ferguson et al., 2018) of the three studies that reported on hospital wide fall rates showed a significant reduction of falls after implementation of the intervention (5.15 vs. 2.49 and 3.58 vs. 2.03 falls per 1,000 patient days, respectively), and the third was underpowered to test for significance (Angel et al., 2016).

### Interprofessional Consultative Team Implementation

#### Facilitators

Several facilitators to intervention implementation and sustainability were highlighted throughout the literature. The majority related to optimizing the team dynamic, using strategies to reinforce frontline staff understanding, and providing unit staff with supportive tools. Team dynamic was described to be optimized by encouraging the development of collegial relationships between team members and ensuring that key clinical roles are included with appropriate governance. For example, it is important to include a clinician knowledgeable about delirium and comfortable consulting with peers as a team leader (Angel et al., 2016) and a skilled geriatrician who can prioritize patient-specific interventions (Marcantonio et al., 2001).

Strategies to maintain frontline staff understanding included providing continuous feedback, creating opportunities for frontline staff to ask questions, education reinforcement related to delirium screening, prevention, and treatment, and obtaining change management support from executive nursing and medicine leaders (Angel et al., 2016; Babine et al., 2013; Bryant et al., 2019; Ferguson et al., 2018; Milisen et al., 2001). Geriatric-specific processes of care were advised to be incorporated into routine clinical care when possible (Bryant et al., 2019), and implementation of the intervention should occur within specialties familiar with the unique needs of older adults, such as geriatric units (Cole et al., 2002). It is beneficial to integrate unit nurses in implementation, as they are frontline clinicians who are able to perform real-time delirium assessments and implement immediate treatment interventions (Ferguson et al., 2018). Finally, including frequent consultations and daily assessments is necessary for the timely detection of health problems, and was noted to improve detection of delirium and allow interprofessional teams to oversee implementation of their recommendations by unit staff (Tarazona-Santabalbina et al., 2019).

Finally, equipping frontline staff with delirium-specific educational, diagnostic, and management resources was frequently identified as a facilitator for success. Unit nurses can be empowered through training and the provision of resources that allow them to confidently prevent, detect, and treat delirium (Ferguson et al., 2018). Bedside tools such as meaningful and easy-to-use protocols or “The Language of Delirium” (a tool that supports detection and evaluation of delirium) can be used to support frontline staffs’ understanding, implementation of and adherence to interventions (Ferguson et al., 2018; Milisen et al., 2001). Finally, it was mentioned to ensure that electronic health records of patients are accessible to all staff involved in their care to streamline assessment and easily identify changes in functional and cognitive status (Ferguson et al., 2018).

#### Barriers

Several barriers to intervention implementation and sustainability were identified. Most related to poor adherence to team recommendations by unit staff, high turnover of interprofessional team members, and difficulty affecting change in processes of care (Cole et al., 2002; Deschodt et al., 2012; Marcantonio et al., 2001). Two studies evaluated recommendation adherence and found that 20% to 30% of treatment recommendations were not adhered to (Deschodt et al., 2012; Marcantonio et al., 2001). Time constraints posed by competing demands and the turnover of delirium team members were also identified as barriers (Angel et al., 2016; Babine et al., 2013; Bryant et al., 2019). One team addressed high rates of turnover by developing a mandatory online module that new clinical staff viewed prior to starting (Bryant et al., 2019).

## Discussion

There is limited literature investigating consultation-based delirium interventions delivered by interprofessional teams and their effectiveness for reducing delirium and its related morbidities. Despite the sparsity and heterogeneity of the literature included in this review, there was notable consistency in interprofessional team structures and core intervention components across the studies. All had various health care disciplines working synergistically, with a physician and nurse role consistently included, to deliver multicomponent interventions the most effective approach to preventing and managing delirium (Teale et al., 2017; Trogrlić et al., 2015).

Several core intervention components consistently emerged across the studies, including; systematic cognitive screening with a validated tool to detect delirium (Babine et al., 2013; Ferguson et al., 2018), pharmacological and non-pharmacological strategies to target modifiable risk-factors, education of unit staff, and ongoing patient consultation from interprofessional delirium team members. In agreement with current evidence these strategies have proven effective for preventing delirium in older adults (Gorski et al., 2017; Hempenius et al., 2013) and often aim to prevent medical and environmental risk factors (Angel et al., 2016; Ferguson et al., 2018; Gorski et al., 2017; Marcantonio et al., 2001). Effective implementation of these strategies was consistently reinforced through educational sessions held to equip unit staff with information and tools required to properly assess, diagnose, and treat delirium to facilitate early intervention and improve patient outcomes (Angel et al., 2016; Ferguson et al., 2018; Milisen et al., 2001). Education was also frequently delivered in real-time as interprofessional teams conducted ongoing consultations and provided patient specific recommendations for delirium management throughout.

Although the interventions included similar core components, their operationalization and evaluation methods varied widely which may underpin the mixed results produced. Most studies reported the effect of the interventions on LOS, delirium incidence, and in-hospital fall rates. Overall, there were some reductions measured but few were significant. Thus, we found some evidence to suggest that interprofessional consultative delirium teams can be effective for reducing delirium and its related comorbidities. This finding is aligned with the analyses of broader inpatient geriatric consultation teams not specific to delirium, which have been unable to conclusively demonstrate the effectiveness of the model despite it being well received by team members, their patients, patient families, nursing managers, and health policy and governmental decision-makers (Deschodt et al., 2016).

Another barrier that may have contributed to the variability and lack of significant findings was the inconsistency in which frontline staff adhered to patient-specific recommendations made by the consultation teams (Cole et al., 2002; Deschodt et al., 2012; Marcantonio et al., 2001). Adherence, or lack thereof, has been found to influence the effectiveness of multicomponent non-pharmacological strategies for delirium prevention in a directly graded fashion (Inouye et al., 2003). Considerable resources and time are required to achieve high levels of staff adherence to intervention protocols, and future consultation-based delirium interventions should address this to optimize their effectiveness. Several strategies to maintain adherence to best clinical practice and facilitate sustainable change have been described throughout this review. Providing continuous support and education to frontline staff, building geriatric processes of care into routine clinical care, and leveraging specialties familiar with the unique needs of older adults could help future researchers mitigate this barrier (Cole et al., 2002). In addition, future efforts should engage clinical staff to further elucidate existing best practices and how they can be achieved while considering the distinct regional, institutional, and cultural context of hospitals.

This review has some limitations. The scope excluded studies published prior to 2000 to emphasize a focus on current literature, and subsequently preliminary, foundational interventions may have been left out. In addition, a critical appraisal of the quality of included studies was not conducted; however, inclusion/exclusion criteria were set to ensure a baseline quality.

### Areas for Future Research

A variety of implementation and evaluation strategies for the included interprofessional consultative delirium team models emerged. The definition and measurement of outcomes, predominantly delirium incidence, severity, and duration, varied widely between studies. The CAM was the most frequently used validated tool to identify delirium; however, it was not used consistently, and screening for delirium was implemented at many different frequencies across the studies. More specific recommendations in clinical guidelines for delirium identification could help establish a standard best practice and reduce the notable heterogeneity across consultation-based delirium interventions. Future efforts should implement increasingly standardized delirium consultation team interventions with aligned outcomes across diverse clinical settings to optimize implementation and evaluation of this model and gain a more comprehensive understanding of its effectiveness.

Furthermore, very few of the studies provided or referenced a detailed intervention protocol, which will hinder guidance and reproducibility for future delirium interventions. Similarly, in instances where the intervention group was compared to usual care, there was rarely a description of the usual care delivered, and therefore, differences in usual care could not be accounted for. To support future efforts to minimize heterogeneity in design, authors should consider reproducibility and include a detailed description of their intervention.

Finally, it is worth noting that the integrated, multicomponent, and interprofessional nature of mobile delirium interventions is foundational to their success but central to the mixed results they produce (Trogrlić et al., 2015). This also makes it challenging to elucidate the effect of individual intervention components on clinical and process outcomes, and subsequently identify which strategies are foundational to the model’s effectiveness.

## Conclusion

Delirium is a highly prevalent disorder that results in very serious mental, physical, and economic consequences for patients, families, and healthcare systems (Angel et al., 2016; Babine et al., 2013). The importance of establishing programs to improve delirium care has gained widespread recognition, and multicomponent interventions are an efficacious and cost-effective approach. Interprofessional delirium consultation teams that provide delirium related education and patient-specific recommendations to frontline staff are an emerging model for the delivery of these multicomponent interventions. We found that this model shows promise to improve delirium care in hospitals and may be effective in reducing delirium incidence. To facilitate implementation, it is important to use effective education strategies, make effort to sustain new practice models, and optimize adherence to recommendations made. While there was consistency in core intervention components across the studies, there was significant variability in operationalization of these components, the selection and ascertainment of outcomes, and results. Further investigation is required to establish the clinical effectiveness of interprofessional consultative delirium team interventions and identify best-practices for their implementation.

## Supplemental Material

sj-pdf-1-jag-10.1177_07334648211018032 – Supplemental material for Effectiveness of Interprofessional Consultation-Based Interventions for Delirium: A Scoping Review Click here for additional data file. Supplemental material, sj-pdf-1-jag-10.1177_07334648211018032 for Effectiveness of Interprofessional Consultation-Based Interventions for Delirium: A Scoping Review by Caitlin Monaghan, Grace Martin, Jason Kerr, Mary-Lynn Peters and Judith Versloot in Journal of Applied Gerontology
